# Ecological momentary assessment in internet gaming disorder – Interactions between stress, negative emotions, craving and gaming behavior

**DOI:** 10.1556/2006.2025.00386

**Published:** 2026-03-19

**Authors:** Alina Killer, Johanna Klar, Stefan Lerch, Julian Koenig, Jochen Kindler, Michael Kaess

**Affiliations:** 1 University Hospital of Child and Adolescent Psychiatry and Psychotherapy, University of Bern, Bern, Switzerland; 2 Clinic and Polyclinic for Child and Adolescent Psychiatry, Psychosomatics and Psychotherapy, University Hospital Cologne, Cologne, Germany; 3 Child and Adolescent Psychiatry, Psychiatry Baselland, Liestal, Switzerland; 4 Department of Child and Adolescent Psychiatry, Center for Psychosocial Medicine, University of Heidelberg, Heidelberg, Germany

**Keywords:** ecological momentary assessment, EMA, internet gaming disorder, stress, coping

## Abstract

**Background and aims:**

Gaming may function as maladaptive coping strategy in youth with internet gaming disorder (IGD). Ecological Momentary Assessment (EMA) enables real-time monitoring of emotions and behavior. This study investigates the temporal dynamics of stress, negative emotions, craving and gaming behavior applying EMA to male youth with IGD during periods of varying gaming intensity and compared to healthy controls (HC).

**Methods:**

29 males diagnosed with IGD, aged 15–25 years, and 26 matched HC were included. Participants underwent diagnostic assessment followed by one week of EMA via smartphone. The group with IGD continued EMA for two additional weeks: one week of unrestricted gaming and one week of restricted gaming. Data on gaming time, stress, negative emotions and craving were collected twice-daily on weekdays and up to sixteen times daily on weekends.

**Results:**

Participants with IGD exhibited significantly higher levels of depression, anxiety, stress, gaming time and craving compared to HC. While gaming time varied between unrestricted and restricted gaming conditions, craving, stress and negative emotions remained stable. In male youth with IGD, gaming was followed by a short-term reduction of stress and negative emotions. However, increased stress and negative emotions were subsequently followed by decreased gaming time.

**Discussion and conclusions:**

This study indicates a complex, bidirectional relationship between gaming, stress and negative emotions. Although gaming provides short-term emotional relief, the association with elevated levels of depression, anxiety and chronic stress highlights its role as maladaptive coping strategy. Conversely, negative emotions and stress do not necessarily seem to increase gaming time.

## Introduction

The research diagnosis internet gaming disorder (IGD) was included in the Diagnostic and Statistical Manual of Mental Disorders (DSM-5) (American Psychiatric Association, 2013) and has a global prevalence of around 2–3% (Stevens, Dorstyn, Delfabbro, & King, 2021, 2023), with higher rates among males, adolescents and young adults (Gao, Wang, & Dong, 2022; Stevens et al., 2021, 2023). In the DSM-5, nine diagnostic criteria were defined by expert consensus including preoccupation, withdrawal, tolerance, unsuccessful control, loss of interest, continuation despite problems, deception, escape negative mood (dysfunctional coping) and jeopardizing opportunities (American Psychiatric Association, 2013). The criterion “using gaming as dysfunctional coping strategy to regulate stress and negative emotions” has been criticized, as it appears to carry less diagnostic value than other criteria (Castro-Calvo et al., 2021; Deleuze et al., 2017; Király, Griffiths, & Demetrovics, 2015; Kuss, Griffiths, & Pontes, 2017) and is also not included in the ICD-11 diagnostic criteria for Gaming Disorder (World Health Organization, 2019). Nonetheless, emotion regulation and coping skills seem to play an important role in the development and maintenance of IGD (Lin, Lin, Lin, Yen, & Ko, 2020; Wichstrøm, Stenseng, Belsky, Von Soest, & Hygen, 2019). Even within the general population, psychological distress has been associated with internet-related addictive behaviors (Huang et al., 2025) and youth with IGD report elevated stress levels and more negative emotions in reaction to stress (Kaess et al., 2017). A recent systematic review indicated that greater difficulties in emotion regulation are associated with the severity of Gaming Disorder (Estupiñá, Bernaldo‐de‐Quirós, Vallejo‐Achón, Fernández‐Arias, & Labrador, 2024). Regarding the neurobiological stress response, differences in IGD have also been observed: While baseline levels of stress hormones seem not to differ in IGD (Killer et al., 2024; Koenig, Thaler, Parzer, Resch, & Kaess, 2019), individuals with IGD show an attenuated cortisol response to acute stress (Kaess et al., 2017). Furthermore, gaming abstinence appears to have a positive effect on stress, as a study found a significant reduction of IGD symptoms, anxiety and stress in a two week longitudinal design (Brailovskaia, Meier-Faust, Schillack, & Margraf, 2022). Theoretical models concerning the development of IGD suggest that the decision to engage in gaming to escape stress or negative moods may represent an important mechanism in the development and maintenance of IGD, as gaming can be perceived as rewarding and therefore reinforce the behavior (Brand, Young, Laier, Wölfling, & Potenza, 2016, 2019; Dong & Potenza, 2014).

Although gaming is considered to serve as short-term coping strategy to deal with stress and negative emotions in individuals with IGD, most studies have focused on longer time intervals spanning several weeks, leaving short-term effects (e.g., within hours) largely unexplored. Ecological momentary assessment (EMA) is ideally suited to investigate short time periods, providing insights into the dynamics of emotions, stress and behavior with high temporal resolution in real time during daily life. Compared to traditional research methods, such as self-report questionnaires, EMA offers improved ecological validity and reduced recollection bias (Shiffman, Stone, & Hufford, 2008). EMA Studies examining the interaction between stress and negative emotions in individuals with IGD during daily life remain scarce. In a Korean study, EMA was applied in a group of youth with IGD and healthy controls (HC), revealing more negative emotions and greater gaming motivation in the group with IGD, which were both positively associated with gaming duration (Kim & Kwon, 2018). Another study used EMA in psychiatric outpatients to assess symptoms of problematic internet use (PIU), anxiety, depression, and smartphone use (Gansner, Nisenson, Lin, Carson, & Torous, 2023). Reduced smartphone use led to worsened depressive and PIU symptoms, indicating a short-term positive effect of smartphone use on mood and PIU symptoms (Gansner et al., 2023). In conclusion, previous studies suggest that youth with IGD or PIU use gaming/their smartphone for mood regulation (Gansner et al., 2023; Kim & Kwon, 2018). While this coping mechanism may offer short-term relief, symptoms of anxiety and depression do not improve in the long term (Gansner et al., 2023).

This study used EMA to investigate the temporal interaction between gaming time, craving, stress and negative emotions in a cross-sectional and longitudinal design. The first aim was to compare how youth with IGD differ from matched HC over the course of one week. The hypothesis was that youth with IGD would report higher levels of self-reported stress, negative emotions, craving and gaming time compared to HC. The second objective was to further investigate these outcomes in the group with IGD for two consecutive weeks, with one week of restricted gaming and one week of unrestricted gaming. Here, it was hypothesized that restricted gaming would increase self-reported stress, negative emotions, and craving compared to unrestricted gaming. The third aim was to assess how gaming, craving, stress and negative emotions are related in short time intervals (hours and days) in youth with IGD. The hypothesis was that higher stress and negative emotions would be followed by increased gaming time and craving, while gaming would reduce stress and negative emotions.

## Methods

### Participants

The sample included 55 male youth aged between 15 and 25 years. Twenty-nine youth with IGD (5–9 DSM-5 IGD criteria) and 26 HC (0–1 DSM-5 IGD criteria) were recruited. The groups were matched for age and educational background. Exclusion criteria for both groups were substance abuse (excluding tobacco), schizophrenia and bipolar I disorder. For the HC group only, additional exclusion criteria were any DSM-5 diagnosis according to the Mini-International Neuropsychiatric Interview (MINI(-KID); Ackenheil, Stotz-Ingenlath, Dietz-Bauer, & Vossen, 1999; Sheehan et al., 2010), meeting more than one DSM-5 IGD criterion, and online gaming use exceeding the Swiss average (1.5 h/day during the week; 2 h/day on weekends; Bernath et al., 2020; Suter et al., 2018). This study was part of a study including an assessment of biomarkers and magnetic resonance imaging (MRI), therefore contraindications for MRI, medication affecting the hypothalamic-pituitary-adrenal (HPA) axis and chronic somatic or neurologic diseases were additional exclusion criteria for both groups. Participants were recruited via public advertisements (e.g. online platforms, schools and universities) in the canton of Bern, Switzerland. IGD subjects were additionally recruited from the outpatient units of the University Hospital of Child and Adolescent Psychiatry and Psychotherapy, Bern, Switzerland. The participants received compensation in the form of vouchers, after completing all scheduled appointments (90CHF for one week, 150CHF for full three-week participation). The group with IGD was further informed about treatment options.

### Study procedure

**Fig. 1.**
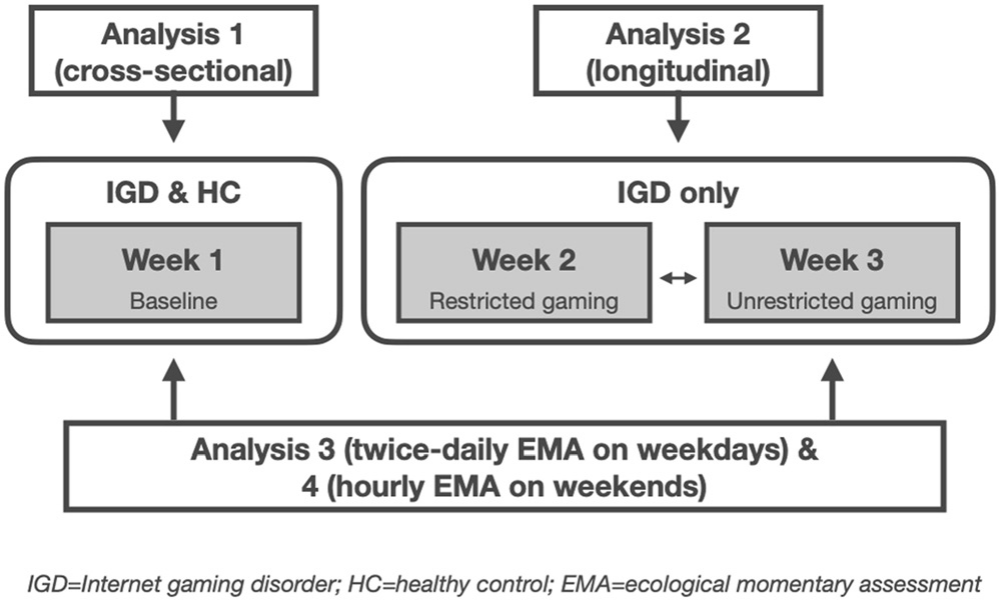
Study design

The study was carried out from January 2019 to January 2023 with simultaneous recruitment of both groups during the whole study period. In a first step, a telephone interview was conducted with all potential participants in which information about the project was provided and inclusion and exclusion criteria were checked. A comprehensive diagnostic assessment was conducted at a first appointment by a trained psychologist and PhD student. During the following week, EMA was applied in the participant's daily routine. Only participants with IGD were followed up for two more weeks, repeating all measures, with one week of unrestricted gaming and one week of restricted gaming (see Fig. 1). The order of the unrestricted and restricted gaming weeks was randomized across participants.

### Diagnostic assessment

The first appointment included the following clinical interviews and questionnaires to assess the severity of IGD and comorbidities. According to the state-of-the-art definition by the American Psychiatric Association (APA), IGD was assessed using the structured clinical interview for IGD diagnosis (DSM-5) (American Psychiatric Association, 2013). To meet diagnostic criteria, participants need to fulfill 5 out of 9 IGD criteria within the last 12 months. Additionally the Video Game Dependency Scale (Computerspielabhängigkeitsskala (CSAS)) was used, a 18-item self-report questionnaire with two questions per DSM-5 IGD criterion (Rehbein, Baier, Kleimann, & Mößle, 2015). Items are answered on a four-point scale and the cut-off for IGD is a sum score above 16 points. The psychometric properties of the CSAS show a good internal consistency (*α* = .95) and test-retest reliability (*r* = .84) (Rehbein et al., 2015). The Mini-International Neuropsychiatric Interview (MINI) covers all common Axis-I psychiatric disorders, listed in the DSM-IV and ICD-10 and was used as a screening instrument for comorbid psychiatric disorders (Ackenheil et al., 1999). In minors, the adjusted interview for children and adolescents (MINI-KID) was used (Sheehan et al., 2010). As anxiety disorders and depression often occur as comorbidities of IGD (Burkauskas et al., 2022; González-Bueso et al., 2018; Ostinelli et al., 2021), the Beck Depression Inventory (BDI-II; Beck, Steer, & Brown, 1996) and the Beck Anxiety Inventory (BAI; Beck, Epstein, Brown, & Steer, 1988) were additionally used to assess depressive symptoms and anxiety levels in more detail. Previous research found elevated chronic stress levels in IGD (Kaess et al., 2017), therefore the Trier Inventory for Chronic Stress (TICS; Schulz & Schlotz, 1999) was used to evaluate participants general stress levels.

### Ecological momentary assessment

To collect dynamic data during the daily routine of participants, EMA was used. The EMA approach was introduced and started after the diagnostic assessment at the first appointment. A study smartphone with the movisensXS app (Movisens GmbH, Karlsruhe, Germany) was handed to each participant. Sampling points were in the morning and evening during weekdays and every hour during the day (8am–11pm) on weekends (total sampling points/subject/week: max. 46). Due to the participants' daily routines, the hourly survey was conducted only on weekends. Morning and evening sampling was started with a button in the app, which participants had to press after waking up and before going to bed. For the hourly sampling on weekends, the participants were reminded with a push message and an acoustic prompt on the study smartphone. Participants were able to postpone the alarm for 5, 10 or 15 minutes. The questions covered gaming time (in min/h), craving, negative emotions and stress (on a visual analogue scale) and the current location (single choice: at home; at work/school; spare time outside the home). Data were stored anonymously on the movisensXS online server with a timestamp for every sampling point and indication of missing data.

### Statistical analyses

Four types of analyses were performed: between-group comparisons (1), comparisons between the unrestricted and restricted gaming week within the group with IGD (2), mixed-effects regressions between daily gaming time, craving, stress and negative emotions during weekdays (3) and time-series analysis of hourly EMA measurements during weekends (4).

For the analyses using time-averaged data (analyses 1 and 2) weekly means per participant for all the measured values were computed. To enable comparisons between hourly measurements during weekends and twice-daily measurements during the week, the hourly data were first averaged into daily values, which were then averaged with the daily measurements during weekdays for each participant. The variables included in the between-group comparisons were gaming time, craving, stress, negative emotions and the root mean square of successive differences (rMSSD) of the reported negative emotions (emotional instability). The rMSSD was computed only for successive hourly measurements during weekends. Group differences between HC group and the group with IGD (analysis 1) were tested using two-sided two-sample *t*-tests. Differences between the unrestricted and restricted gaming conditions in the group with IGD (analysis 2) were analyzed using linear mixed effects models with fixed effect for the condition and a random intercept for each participant.

For analyses 3 and 4, data from the baseline and unrestricted gaming week of the group with IGD were used to examine temporal patterns in the behavior and emotional experiences of the participants. The twice-daily relationships on weekdays (analysis 3) were analyzed using linear mixed models with random intercepts per participant. A first model tested whether the reported daily gaming time is predicted by the morning levels of stress, negative emotions and craving. It was further checked if the daily craving is predicted by the morning levels of stress, negative emotions or daily gaming time. A second model tested whether overall daily stress or negative emotions are predicted by gaming time or craving, when corrected for the respective morning levels.

The hourly EMA time-series during the weekends (analysis 4) were analyzed using multilevel linear regressions, with random intercepts for each participant (level 1) and for each continuous segment of hourly EMA measurements within each participant (level 2). To account for the autocorrelation of the time series, the residuals within each segment were modelled using an autoregressive AR (1) correlation structure. Again, a first model tested the possible effect of gaming time/craving in the previous hour on stress/negative emotions, and a second model tested the effect of the level of stress/negative emotions on the gaming time/craving in the subsequent hour. In a third model, the effect of gaming time/craving in the previous hour on craving/gaming time in the subsequent hour was tested. To check if the current location could be a potential confounder, a sensitivity analysis was performed which restricted the time-series analysis to those time points where the participants reported being at home.

To check whether the results of analyses 3 and 4 differ between the group with IGD and HC group, the analyses including both groups, including an interaction with the group variable were rerun.

The analyses were run using Stata/SE 18.5 (StataCorp, College Station, TX) and all tests were two-sided with significance level set at *α* = 0.05.

### Ethics

The study procedures were carried out in accordance with the Declaration of Helsinki and approved by the local ethics committee of the University of Bern (KEK 2018-01604). All participants provided written informed consent. In case of minors, consent was additionally provided by parents.

## Results

The total sample of the cross-sectional part of the study included 55 participants with 29 fulfilling the IGD criteria (mean = 6.5; range: 5–9) and 26 HC (mean = 0.4; range 0–1). The groups were matched with no significant differences in age (*p* = .31) or education (*p* = .14, see Table 1). Five of the 29 participants with IGD (17.24%) met the criteria for generalized anxiety disorder, attention deficit hyperactivity disorder or agoraphobia according to the MINI(-KID) screening. The questionnaires showed significant differences between the groups with higher scores in the group with IGD in CSAS (*p* < .001), BDI-II (*p* = .003), BAI (*p* = .02), and TICS (*p* = .02, see Table 1), indicating more problematic gaming behavior as well as higher stress, depressiveness and anxiety in IGD compared to HC. Due to poor compliance, four individuals of the group with IGD dropped out after the first week of measurements, therefore only 25 participants with IGD were included in the longitudinal part of the study.

**Table 1.** Sample – sociodemographic and clinical characteristics (cross-sectional)

	IGD^a^	HC^b^	Group comparison
N (sample size)	29	26	
Age (in years, mean)	19.8	20.7	t(53) = 1.02, *p* = .31
Education^c^ (ISCED 2/ISCED 3/BSc/MSc)	5/23/1	2/19/5	*p* = .14
CSAS^d^ (sum score, mean)	20.1	3.6	t(53) = −8.78, *p* < .001
BDI-II^e^ (mean)	9.1	4.3	t(53) = −3.14, *p* = .003
BAI^f^ (mean)	10.1	6.2	t(53) = −2.37, *p* = .02
TICS^g^ (mean)	74.1	51.2	t(53) = −2.39, *p* = .02

^a^Internet gaming disorder;

^b^Healthy control;

^c^International Standard Classification of Education (ISCED);

^d^Computerspielabhängigkeitsskala;

^e^Beck Depression Inventory;

^f^Beck Anxiety Inventory;

^g^Trier Inventory for Chronic Stress.

### Between group comparison of EMA data (analysis 1, cross-sectional)

No significant difference between groups was found in the EMA response rate (*p* = .18, see Table 2). The analyses of EMA data showed a significant difference in the weekly gaming time (*p* < .001) and craving (*p* < .001) between groups, but no differences in experienced stress (*p* = .50), negative emotions (*p* = .68) or emotional instability (rMSSD, *p* = .50).

**Table 2.** Between group comparison of EMA data (cross-sectional)

	IGD^a^	HC^b^	Group comparison
Response rate (mean %)	86.0	77.1	t(53) = −1.34, *p* = .18
Gaming time (mean h per week)	23.5	4.0	t(53) = −4.71, *p* < .001
Craving (mean/median)	36.1/34.7	13.5/12.6	t(53) = −5.25, *p* < .001
Stress (mean/median)	22.1/22.9	24.5/20.2	t(53) = 0.68, *p* = .50
Neg. Emotions (mean/median	21.2/23.2	22.6/19.5	t(53) = 0.41, *p* = .68
Emotional instability (rMSSD^c^ mean)	12.0	11.0	t(51) = −0.68, *p* = .50

^a^Internet gaming disorder;

^b^Healthy control;

^c^Root mean square of successive differences (rMSSD) was computed only for successive hourly measurements of negative emotions during the weekend.

### Comparison of restricted vs. unrestricted gaming in the group with IGD (analysis 2, longitudinal)

Within the three weeks of the longitudinal part of the study, the participants with IGD showed a significant difference in response rate, with lower response rates in the last two weeks compared to the first week (*p* < .001). The within-subject analyses of EMA data showed a significant difference between gaming conditions in gaming time (*p* < .01), but no significant differences in craving (*p* = .63), experienced stress (*p* = .19), negative emotions (*p* = .22) and emotional instability (*p* = .21). The lowest amount of stress, negative emotions, and emotional instability was reported in the restricted gaming week, however with no significant differences between groups (see Table 3).

**Table 3.** Between gaming conditions comparison of EMA data in the group with IGD (longitudinal)

	Baseline *N* = 29	Unrestricted gaming *N* = 25	Restricted gaming *N* = 25	Comparison restricted vs. unrestricted gaming
Response rate (mean %, per week)	86.0	75.4	68.8	*p* = .07
Gaming time (mean, h)	23.5	18.8	0.4	*p* < .01
Craving (mean, median)	36.1 (34.7)	43.0 (45.2)	40.6 (31.8)	*p* = .63
Stress (mean, median)	22.1 (22.9)	24.0 (25.4)	20.9 (16.3)	*p* = .19
Neg. Emotions (mean, median)	21.2 (23.2)	21.0 (21.7)	18.1 (19.7)	*p* = .22
Emotional Instability (rMSSD^a^, mean)	12.0	11.6	8.9	*p* = .21

^a^root mean square of successive differences (rMSSD) was computed only for successive hourly measurements of negative emotions during the weekend.

### Twice-daily EMA time-series analysis on weekdays (analysis 3)

For male youth with IGD, the twice-daily EMA time-series analysis revealed significantly higher daily craving following more negative emotions in the morning (Coefficient = .33; 95% CI [.12; .53]; SD = .10; *p* = .002). Additionally, increased daily gaming time was followed by higher daily craving reports in the evening (Coefficient = 1.64; 95% CI [.65; 2.64]; SD = 0.51; *p* = .001). There was no statistically significant effect of daily gaming time on stress (*p* = .25) and negative emotions (*p* = .88), daily craving on stress (*p* = .73) and negative emotions (*p* = .96), stress on daily gaming time (*p* = .55) and daily craving (*p* = .57), negative emotions (*p* = .27) and craving (*p* = .08) on daily gaming time.

When including the HC group in the analysis a significant group interaction (*p* = 0.03) was found for the association of daily gaming time on stress: While the group with IGD reported slightly less stress with increased daily gaming time (Coefficient = −.64; 95% CI [−1.66; .39]; SD = .52.; *p* = .22) the HC group reported slightly higher stress with increased daily gaming time (Coefficient = 1.64; 95% CI [−.19; 3.47]; SD = .93; *p* = *.08)*. However, neither of the individual effects reached statistical significance. No further group interactions were found.

### Hourly EMA time-series analysis on weekends (analysis 4)

**Fig. 2.**
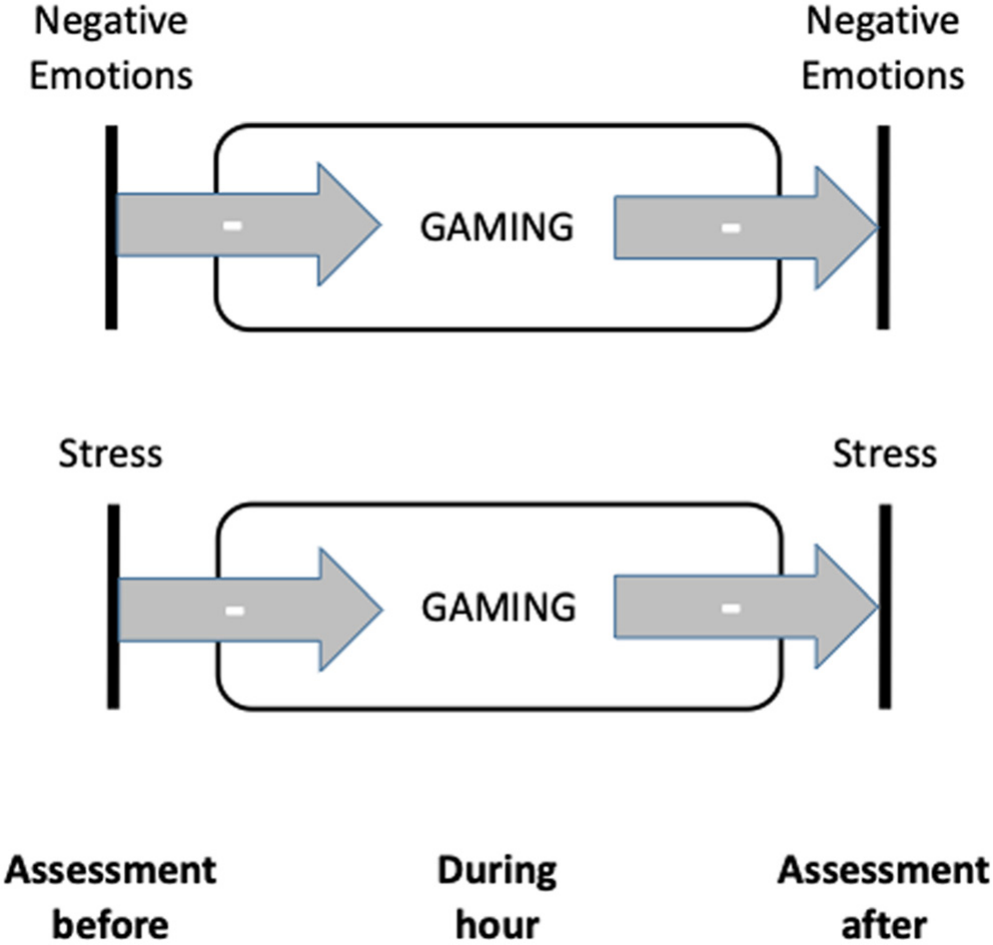
Hourly EMA time-series analysis on weekends

For male youth with IGD, the hourly EMA time-series analysis showed a significant reduction of stress (*p* = .008) and negative emotions (*p* = .004) at the subsequent EMA sampling time point after gaming (see Table 4 and Fig. 2). Further, a significant decrease in gaming time after experiencing stress (*p* = .001) and negative emotions (*p* = .001; see Table 4 and Fig. 2) was detected. No significant effects were found for the interaction of stress, negative emotions and gaming time with craving and vice versa (see Table 4). No significant group interactions were found in the analysis including the HC group.

**Table 4.** Hourly effects of gaming, craving, stress and negative emotions on weekends in the group with IGD

	Coefficient	Standard error	*Z*	95% Confidence interval	*P*
Gaming → Stress	−.05	.02	−2.65	−.09 to −.01	.008
Gaming → Neg. emotions	−.05	.02	−2.89	−.08 to −.01	.004
Stress → Gaming	−.14	.04	−3.31	−.22 to −.06	.001
Neg. emotions → Gaming	−.16	.05	−3.25	−.26 to −.07	.001
Stress → Craving	−.02	.04	−.55	−.10 to −.06	.58
Craving → Stress	.02	.03	.73	−.04 to −.08	.47
Neg. emotions → Craving	.05	.05	1.04	−.05 to −.15	.30
Craving → Neg. emotions	−.001	.02	−.06	−.05 to −.04	.95
Gaming → Craving	−.02	.04	−.57	−.10 to −.05	.57
Craving → Gaming	.05	.03	1.50	−.02 to −.11	.13

## Discussion

This study presents a comprehensive analysis of the interaction between stress, negative emotions, craving and gaming behavior in male youth with IGD compared to HC, further addressing different gaming conditions in those with IGD. The multifaceted approach, including cross-sectional and longitudinal designs with EMA, offers valuable insights into the complex dynamics of IGD. Applying EMA enabled the assessment of short-term changes in negative emotions, stress and craving depending on the gaming behavior and to assess whether those effects occurred within hours or days.

### Comparison of IGD and HC at baseline (cross-sectional)

The participants with IGD showed significantly higher levels of chronic stress, anxiety and depression compared to HC, indicating greater psychological distress in IGD. This finding aligns well with previous research that has identified depression and anxiety disorders as the most prevalent comorbidities of IGD (Burkauskas et al., 2022; González-Bueso et al., 2018; Ostinelli et al., 2021). However, comorbid disorders such as depression or anxiety were an exclusion criterion for the HC group only, which may have inflated the observed group differences. Interestingly, in contrast to the questionnaires, the EMA data showed only a difference in craving between the groups but not the expected differences in stress, negative emotions and emotional instability. Nonconvergence between self-report questionnaires and EMA have frequently been reported and have various underlying factors (Stinson, Liu, & Dallery, 2022). Most importantly, questionnaires investigate long-term intervals covering days to weeks, whereas EMA captures short-term periods of minutes to hours. Another factor might be reduced compliance to answer the demanded EMA survey in stressful or emotionally difficult situations, causing an underreporting of these states.

### Comparison of restricted vs. unrestricted gaming (longitudinal)

In contrast to our hypothesis craving, stress, negative emotions and emotional instability were not significantly higher during the restricted gaming week, indicating that in IGD craving is less about the amount of gaming and more about the individual's relationship with the gaming activity itself. This finding supports the Delphi study, where withdrawal symptoms were not confirmed as a main criterion for IGD diagnosis (Castro-Calvo et al., 2021). Another study found withdrawal symptoms declined already after one day (Yen, Lin, Wu, & Ko, 2022). Therefore, in the weekly average a short-term increase of craving may not have been detectable. Stress, negative emotions and emotional instability were lowest during the restricted gaming week. Even though the symptom reduction during the restricted gaming week was not significant, this might already indicate tendencies of symptom improvement after one week of restricted gaming. Brailovskaia et al. found significant effects after two weeks of gaming abstinence (Brailovskaia et al., 2022), therefore the regarded time period of one week might have been too short to observe significant effects. Even though it is not fully understood during which time frame symptoms might improve, it is an important finding - also for a clinical context - that experienced stress, negative emotions and craving seem not to worsen during the first week of gaming withdrawal.

### Twice-daily and hourly EMA time-series analyses: divergent patterns

The hourly EMA time-series analysis on weekends revealed a significant reduction in stress and negative emotions at the subsequent EMA sampling time point after gaming in the group with IGD. This short-term relief could indicate that gaming functions as a temporary coping strategy for emotional distress. The twice-daily EMA analysis on weekdays did not reveal similar effects, indicating only a very short-term improvement that does not persist throughout the day. However, a significant group difference was still found in the twice-daily analysis on weekdays (gaming leading to less stress in the group with IGD, but more stress in the HC group). Regarding PIU, Gansner et al., showed a reduction of depressive and anxiety symptoms with higher smartphone usage over the course of days (Gansner et al., 2023). A short-term relief might be preferred over long-term negative consequences, such as chronic stress, anxiety and depression. Especially in later stages of addictive disorders, this negative reinforcement seems to play an important role for maintaining game-use despite its negative consequences (Brand et al., 2025). This is supported by a meta-analysis showing a consistent relation between IGD, dysfunctional risk evaluation, reward processing and decision-making (Yao, Zhang, Fang, Liu, & Potenza, 2022). Alterations of critical neuronal networks, such as the fronto-striatal network, might underlie the dysfunctional decision-making favouring short-term reward in IGD (Klar et al., 2024; Punia & Balodis, 2019). The dopaminergic reward system is highly involved in the reward learning processes, as well as in coping with stress (Baik, 2020). Alterations in the dopaminergic reward system seem to enhance reward sensitivity and therefore promote the development of addictive behaviors (Baik, 2020; Ironside, Kumar, Kang, & Pizzagalli, 2018; Popescu, Marian, Drăgoi, & Costea, 2021).

The observed decrease in gaming time following hours of heightened stress and negative emotions suggests a complex, bidirectional relationship. This result contrasts with the findings of Kim & Kwon, who reported increased gaming time with experienced negative emotions (Kim & Kwon, 2018). However, they investigated substantially longer timeframes, supporting the notion that temporal resolution is an important factor when considering emotion-behavior interactions in IGD. Our result might also reflect external factors such as homework or other responsibilities, which may cause stress and negative emotions while limiting time available for gaming. As craving did not lead to higher gaming time (in the twice-daily and hourly analyses), this could imply that some degree of inhibitory control remained intact in this sample. Previous research underlined disturbed craving regulation as core mechanism in IGD (Zhang et al., 2021). This finding could not be reproduced in this study, as craving showed no significant interactions for the group with IGD in the hourly analysis on weekends. Nevertheless, the twice-daily EMA analysis on weekdays revealed a significant increase of craving co-occurring with high daily gaming time and following negative emotions. As this effect was not evident in the hourly analysis it remains unclear whether the effect is limited to daily timescales or if differences between the weekend and weekdays might play a role. However increased craving after gaming has been reported before (Dong, Wang, Du, & Potenza, 2017).

### Strengths and limitations

The main strength of this study was the application of EMA in the daily life of male youth with IGD and a well-matched control group, as well as the inclusion of a longitudinal arm with different gaming conditions. The twice-daily and hourly EMA design allowed measurements of short-term interactions and enabled the assessment of whether effects occur within the time frame of hours or days. However, due to the participants' everyday routines, daily effects were only measured during weekdays and hourly effects only on weekends. This raises the question of whether effects can be attributed specifically to the measured time frame or whether differences between weekdays and weekends might have played a major role. Since participants could have a more structured daily routine during the week, as well as less time for gaming, effects might be less pronounced during the week. Considering that for the significant findings of the twice-daily and hourly analyses the group interactions with the HC group were not significant, results might indicate general mechanisms and might not be specific to IGD. Overall, EMA response rates were satisfactory. Nevertheless, there was a response fatigue with increased duration of the survey leading to lower response rates after the first week in participants with IGD. Since the conditions were randomized between weeks, the influence of the response rate on the results was controlled and there was no significant difference in the response rate between unrestricted and restricted gaming conditions. Furthermore, one limiting factor of this study is the sampling period of only one week in the HC group and only one week of restricted gaming in the group with IGD, which may have been too short to reveal effects. Due to our exclusion criteria only the group with IGD showed comorbid disorders. It is therefore unclear at what point effects can be attributed solely to IGD. However, comorbid disorders are very common in IGD (Burkauskas et al., 2022; González-Bueso et al., 2018; Ostinelli et al., 2021). Furthermore, as we did not adjust for help-seeking and/or treatment status among participants with IGD in our analyses, the influence of these variables cannot be completely ruled out. Since the sample size was quite small and included only male participants with IGD, it is important to consider a larger sample size in future studies, as well as the inclusion of female participants, to increase statistical power and validity.

### Implications for treatment

The findings of this study have implications for the treatment of IGD. Psychotherapeutic interventions focusing on stress management, emotional regulation and alternative coping strategies might be particularly beneficial for individuals with IGD. Given the significant psychological distress and comorbidities seen in the group with IGD, such as increased chronic stress and symptoms of depression and anxiety, an assessment of these symptoms and, if present, holistic treatment approach, is crucial. Our study was able to show that even with restricted gaming, an increase in stress and negative emotions is unlikely within the first week. These are promising findings regarding the reduction of gaming time in patients.

### Future research

Overall, the results warrant further exploration into whether gaming serves as a cause or a consequence of emotional dysregulation in IGD, as well as the role that craving plays in this context. Future research should continue to explore the nuanced relationship between gaming behavior, craving and emotional states in IGD. Longitudinal studies with larger sample sizes and diverse populations could provide further insights into the causality and direction of these relationships. Additionally, investigating the potential moderating factors, such as personality traits and social support, could enhance our understanding of the psychopathology in IGD. The inclusion of other disorders concerning online behavior, such as social media disorder, could be considered to examine whether these findings can be extended to other forms of internet addiction.

## Conclusions

To our knowledge, this study is the first to show a reduction of stress and negative emotions within hours after gaming. Thus, our results support the clinical notion that young patients with IGD use gaming as a dysfunctional coping strategy to efficiently reduce stress in the short term. However, this seems not to be sustainable on the long term as shown by the higher anxiety, depression and stress levels compared to HC. Conversely, negative emotions and stress do not necessarily seem to increase gaming time. Symptoms as craving, stress and negative emotions did not worsen during the evaluation of one week of restricted gaming, indicating that, if any, only very short-term withdrawal symptoms might occur.
